# Aneurysm treatment within 6 h versus 6–24 h after rupture in patients with subarachnoid hemorrhage

**DOI:** 10.1177/23969873231173273

**Published:** 2023-05-01

**Authors:** Mervyn DI Vergouwen, Menno R Germans, René Post, Maud A Tjerkstra, Bert A Coert, Gabriel JE Rinkel, William Peter Vandertop, Dagmar Verbaan

**Affiliations:** 1 Department of Neurology and Neurosurgery, UMC Utrecht Brain Center, University Medical Center Utrecht, Utrecht University, Utrecht, The Netherlands; 2 Department of Neurosurgery, Clinical Neuroscience Center, University Hospital Zurich, Zurich, Switzerland; 3 Department of Neurosurgery, Amsterdam UMC Location University of Amsterdam, Amsterdam, The Netherlands; 4 Amsterdam Neuroscience, Neurovascular Disorders, Amsterdam, The Netherlands

**Keywords:** Subarachnoid hemorrhage, aneurysm, treatment, clipping, endovascular treatment, coiling, timing

## Abstract

**Background:**

The risk of rebleeding after aneurysmal subarachnoid hemorrhage (aSAH) is the highest during the initial hours after rupture. Emergency aneurysm treatment may decrease this risk, but is a logistic challenge and economic burden. We aimed to investigate whether aneurysm treatment <6 h after rupture is associated with a decreased risk of poor functional outcome compared to aneurysm treatment 6–24 h after rupture.

**Methods:**

We used data of patients included in the ULTRA trial (NCT02684812). All patients in ULTRA were admitted within 24 h after aneurysm rupture. For the current study, we excluded patients in whom the aneurysm was not treated <24 h after rupture. We calculated crude and adjusted risk ratios (aRR) with 95% confidence intervals using Poisson regression analyses for poor functional outcome (death or dependency, assessed by the modified Rankin Scale) after aneurysm treatment <6 h versus 6–24 h after rupture. Adjustments were made for age, sex, clinical condition on admission (WFNS scale), amount of extravasated blood (Fisher score), aneurysm location, tranexamic acid treatment, and aneurysm treatment modality.

**Results:**

We included 497 patients. Poor outcome occurred in 63/110 (57%) patients treated within 6 h compared to 145/387 (37%) patients treated 6–24 h after rupture (crude RR: 1.53, 95% CI: 1.24–1.88; adjusted RR: 1.36, 95% CI: 1.11–1.66).

**Conclusion:**

Aneurysm treatment <6 h is not associated with better functional outcome than aneurysm treatment 6–24 h after rupture. Our results do not support a strategy aiming to treat every patient with a ruptured aneurysm <6 h after rupture.

## Introduction

In patients with aneurysmal subarachnoid hemorrhage (SAH), rebleeding of the aneurysm increases the risk of poor functional outcome.^1^ The recently published ULTRA trial showed that ultra-early, short-term treatment with tranexamic acid (TXA) does not result in a statistically significant reduction in the risk of rebleeding, and does not improve functional outcome.^2,3^ Therefore, other strategies or interventions are needed that reduce the risk of rebleeding and improve functional outcome after SAH. Since the risk of rebleeding is the highest in the first hours after rupture,^4^ emergency aneurysm treatment within 6 h after rupture seems an attractive strategy.^5^ However, emergency aneurysm treatment may also increase the risk of per-procedural complications including rebleeding and ischemia.^6–12^ In addition, emergency aneurysm treatment is a logistic and economic burden and increases the workload of neurosurgical and neuroradiological teams considerably. We aimed to investigate whether emergency aneurysm treatment within 6 h after rupture is associated with a decreased risk of poor functional outcome compared to patients treated between 6 and 24 h.

## Methods

This was a post hoc study of the ULTRA trial (NCT02684812), which was a multicenter randomized, controlled trial that included adult patients with spontaneous CT-proven subarachnoid hemorrhage in 8 treatment centers and 16 referring hospitals in the Netherlands between July 2013 and July 2019.^2^ In ULTRA, only patients were included with a most recent rupture ⩽24 h. For the current study, exclusion criteria were: (1) no aneurysm identified; (2) no aneurysm treatment; and (3) aneurysm treatment more than 24 h after rupture.

The following variables were collected: age, sex, clinical condition on admission according to the WFNS score,^13^ amount of extravasated blood according to the Fisher et al.^14^ score, use of platelet inhibitors on admission, use of anticoagulants on admission, aneurysm location, time of rupture, time of presentation at emergency department, time of first CT diagnosis, TXA treatment, any rebleeding (both before and after randomization), treatment modality, time of aneurysm treatment, per-procedural rupture, thromboembolic complications during endovascular treatment, delayed cerebral ischemia (DCI), modified Rankin Scale (mRS) score, and case-fatality after 6 months.

### Primary and secondary outcomes

The primary outcome measure was poor functional outcome, defined as a mRS of 3–6 months after rupture. The secondary outcome measure was case-fatality.

### Analyses

Patients were divided into two groups: those with aneurysm treatment within 6 h and those with aneurysm treatment between 6 and 24 h after rupture. Baseline characteristics, in-hospital complications, and outcome characteristics were described for both groups.

First, we calculated risk ratios (RR) with 95% confidence intervals (CI) for the primary and secondary outcome measures with the group of patients treated 6–24 h after rupture as the reference group. Adjusted risk ratios (aRR) were calculated using Poisson regression with adjustments for the following predefined variables: age, sex, WFNS score, Fisher score (Fisher 1–3 vs 4), location of the aneurysm (posterior vs anterior circulation), TXA treatment (as treated), and aneurysm treatment modality.

Second, we performed additional analyses to adjust for potential sources of bias or confounding that may lead to worse outcome in the emergency treatment group. Since rebleeding shortly after rupture may increase urgency to treat the ruptured aneurysm as soon as possible, whereas these patients may have been treated in the 6–24 h interval if rebleeding had not occurred, this bias by indication leads to a larger proportion of patients with a poor prognosis in the emergency treatment group. To overcome this source of bias, we performed an additional analysis for the primary outcome measure in which all patients with rebleeding and aneurysm treatment within 6 h were re-categorized to the group of patients treated 6–24 h after rupture. Third, we performed an analysis to investigate whether emergency aneurysm treatment itself impacts functional outcome irrespective of rebleeding. For this analysis, we excluded all patients with rebleeding of the aneurysm, since rebleeding is an important cause of poor functional outcome and a major confounder in the main analysis.

Finally, we performed a subgroup analysis for type of aneurysm treatment for the primary outcome.

## Results

**Figure 1.**
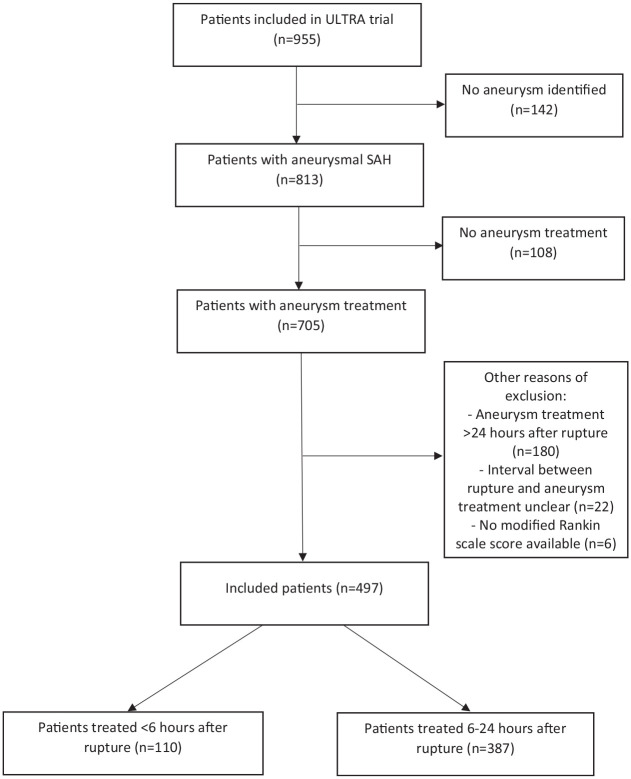
Flowchart.

We included 497 patients (aneurysm treatment within 6 h after rupture: *n* = 110; aneurysm treatment 6–24 h after rupture: *n* = 387) (Figure 1). Baseline characteristics are shown in Table 1. A total of 54/110 (49%) patients with aneurysm treatment within 6 h had a WFNS score on admission of 4 or 5, compared to 121/387 (31%) patients with aneurysm treatment 6–24 h after rupture. Presentation at the emergency department between 6 AM and 6 PM occurred in 93/110 (85%) patients with aneurysm treatment within 6 h, compared to 162/387 (42%) patients with aneurysm treatment 6–24 h after rupture. Aneurysm treatment between 6 PM and midnight occurred in 31/110 (28%) patients with aneurysm treatment within 6 h, compared to 48/387 (12%) patients with aneurysm treatment 6–24 h after rupture. Aneurysm treatment during nighttime occurred in 8/497 (2%) patients of the total cohort.

**Table 1.** Baseline characteristics according to time of aneurysm treatment.

	All patients (*n* = 497)	Aneurysm treatment <6 h after rupture (*n* = 110)	Aneurysm treatment 6–24 h after rupture (*n* = 387)
Median age (IQR)	57 (49–65)	56 (48–64)	57 (49–66)
Female sex (*n*, %)	353 (71)	74 (67)	279 (72)
WFNS score on admission (*n*, %)			
1	184 (37)	27 (25)	157 (41)
2	104 (21)	21 (19)	83 (21)
3	29 (6)	8 (7)	21 (5)
4	111 (22)	34 (31)	77 (20)
5	64 (13)	20 (18)	44 (11)
Unknown	5 (1)	–	5 (1)
Fisher score on admission (*n*, %)			
1	–	–	–
2	22 (4)	2 (2)	20 (5)
3	157 (32)	24 (22)	133 (34)
4	318 (64)	84 (76)	234 (60)
Use of platelet aggregation inhibitors on admission (*n*, %)	51 (10)	10 (9)	41 (11)
Use of anticoagulants on admission (*n*, %)	10 (2)	5 (5)	5 (1)
TXA treatment (as treated population) (*n*, %)	243 (49)	44 (40)	199 (51)
Aneurysm circulation (*n*, %)			
Anterior	424 (85)	91 (83)	333 (86)
Posterior	72 (14)	19 (17)	53 (14)
Unclear	1 (<1)	–	1 (<1)
Time of arrival in hospital (*n*, %)			
00.00–05.59 h	73 (15)	4 (4)	69 (18)
06.00–11.59 h	99 (20)	35 (32)	64 (17)
12.00–17.59 h	156 (31)	58 (53)	98 (25)
18.00–23.59 h	160 (32)	12 (11)	148 (38)
Unknown	9 (2)	1 (1)	8 (2)
Aneurysm treatment modality (*n*, %)			
Endovascular	388 (78)	89 (81)	299 (77)
Clipping	109 (22)	21 (19)	88 (23)
Time of aneurysm treatment (*n*, %)			
00.00–05.59 h	8 (2)	3 (3)	5 (1)
06.00–11.59 h	152 (31)	9 (8)	143 (37)
12.00–17.59 h	258 (52)	67 (61)	191 (49)
18.00–23.59 h	79 (16)	31 (28)	48 (12)

In-hospital complications and outcome characteristics are shown in Table 2. Any rebleeding occurred in 29/110 (26%) patients with aneurysm treatment within 6 h, compared to 49/387 (13%) patients with aneurysm treatment 6–24 h after rupture.

**Table 2.** In-hospital complications and outcomes according to time of aneurysm treatment.

	All patients (*n* = 497)	Aneurysm treatment <6 h after rupture (*n* = 110)	Aneurysm treatment 6–24 h after rupture (*n* = 387)
Any rebleeding* (*n*, %)	78 (16)	29 (26)	49 (13)
Per-procedural rupture (*n*, %)	53 (11)	27 (25)	26 (7)
DCI (*n*, %)	137 (28)	36 (33)	101 (26)
Thromboembolic complications during endovascular treatment (*n*, %)	46/388 (12)	9/89 (10)	37/299 (12)
Poor functional outcome (*n*, %)	208 (42)	63 (57)	145 (37)
Case-fatality (*n*, %)	83 (17)	29 (26)	54 (14)

* Any rebleeding, both prior and after randomization.

### Primary and secondary outcome measures

Poor outcome occurred in 63/110 (57%) of patients treated within 6 h compared to 145/387 (37%) of patients treated 6–24 h after rupture (crude RR: 1.53, 95% CI: 1.24–1.88; adjusted RR: 1.36, 95% CI: 1.11–1.66). Case-fatality occurred in 29/110 (26%) of patients treated within 6 h compared to 54/387 (14%) of patients treated 6–24 h after rupture (crude RR: 1.89, 95% CI: 1.27–2.81; adjusted RR: 1.57, 95% CI 1.07–2.33).

### Secondary analysis: All patients with rebleeding in 6–24 h group

Rebleeding occurred in 29 patients who had aneurysm treatment within 6 h after rupture. If these patients were added to the group of patients with aneurysm treatment between 6 and 24 h after rupture, the crude RR for poor functional outcome for treatment within 6 h after rupture was 1.19 (95% CI: 0.92–1.53) and the adjusted RR 1.03 (95% CI: 0.82–1.29).

### Tertiary analysis: Exclusion of patients with rebleeding

For this analysis, 78 patients with any rebleeding were excluded. Poor functional outcome occurred in 39/81 patients (48%) treated within 6 h and in 113/338 (33%) patients treated between 6 and 24 h after rupture (crude RR: 1.44, 95% CI: 1.10–1.89; adjusted RR: 1.23, 95% CI: 0.96–1.58).

### Subgroup analysis: Type of aneurysm treatment

In patients with endovascular aneurysm treatment, poor functional outcome occurred in 48/89 patients (54%) treated within 6 h and in 107/299 (36%) patients treated between 6 and 24 h after rupture (crude RR: 1.51, 95% CI: 1.18–1.93; adjusted RR: 1.34, 95% CI: 1.06–1.70). In patients with neurosurgical aneurysm treatment, poor functional outcome occurred in 15/21 patients (71%) treated within 6 h and in 38/88 (43%) patients treated between 6 and 24 h after rupture (crude RR: 1.65, 95% CI: 1.15–2.37; adjusted RR: 1.45, 95% CI: 1.01–2.08).

## Discussion

This study showed that aneurysm treatment within 6 h after rupture is not associated with better functional outcomes compared to treatment between 6 and 24 h. In additional analyses, in which we adjusted for potential sources of bias or confounding at the expense of emergency aneurysm treatment, treatment within 6 h after rupture was also not associated with better outcomes. Similar effects were observed in patients with endovascular and neurosurgical aneurysm treatment.

A randomized trial at the end of the 1980s found no difference in outcome in SAH patients with aneurysm treatment within 3 days after rupture versus delayed aneurysm treatment more than 7 days after rupture.^15^ Since then, timing of aneurysm treatment has shifted from a delayed phase to an early phase within 72 h after rupture. More recent studies focused on aneurysm treatment within 72 h after rupture and found that aneurysm treatment within 24 h is not associated with better outcomes than aneurysm treatment 24–72 h after rupture.^16–18^ Nevertheless, treatment within 24 h is common practice in most centers, and supported by the guidelines of the American Heart Association, which recommend aneurysm treatment as early as feasible.^19^

Although aneurysm treatment within 6 h after rupture may theoretically decrease the risk of rebleeding and hereby increase the chances of good functional outcome, our data do not show better outcomes in patients treated within 6 h. Instead, we found a higher proportion of poor functional outcome and case-fatality in patients treated within 6 h compared to 6–24 h after rupture. Although bias by indication may play a role, similar findings were found after adjustment for several baseline characteristics including clinical condition on admission and Fisher scale. A potential explanation for the worse outcomes in the group of patients treated within 6 h, may be that re-rupture, including per-procedural rupture, of the aneurysm was more common in the group of patients treated within 6 h compared to patients with aneurysm treatment 6–24 h after rupture.^12^ However, also in our secondary and tertiary analyses, in which we accounted for the higher proportion of rebleeding in the group of patients treated within 6 h, treatment within 6 h was not associated with better outcomes. A second explanation may be that emergency aneurysm treatment, or related general anesthesia, increases the extent of early brain injury and the risk of DCI and hereby results in worse outcomes. This second explanation is supported by the observation of worse outcomes in patients treated within 6 h after rupture in our tertiary analysis, in which all patients with rebleeding were excluded.

For the purpose of the ULTRA trial, no data were collected on reasons for treatment within 6 h or after 6–24 h. However, the data on time of presentation to the emergency department and time of aneurysm occlusion show that the majority of patients who were treated within 6 h presented to the emergency department between 6 AM and 6 PM, and that most patients who had aneurysm treatment 6–24 h after the bleeding presented to the emergency department in the evening or during the night. Since only very few patients were treated during the night, these data suggest that time of presentation to the emergency department during evening or night times was an important reason to delay aneurysm treatment to the next day.

A strength of our study is that we used data from a recently completed randomized controlled trial, in which data were prospectively recorded. As a result, we only had small numbers of missing data. In addition, because of the ULTRA research question, we had detailed data on timing of rupture and aneurysm treatment. Since this was a post-hoc study of the ULTRA trial, the research nurse who performed the outcome assessment 6 months after rupture was not aware of the current research question. Since all patients included in the ULTRA trial had bleeding of the aneurysm in the previous 24 h and most instances of aneurysmal rebleeding occur within the first 24 h after rupture, this cohort was suitable for the current research question. A limitation of our study is that the patients were not randomized for timing of aneurysm treatment, so the results are prone to bias by indication. Aneurysm treatment may have been prioritized because of a poor clinical condition or because of aneurysmal rebleeding and an inherent neurological worsening. This will have resulted in differences in baseline characteristics between the two groups and the higher proportion of rebleeding in the patients with aneurysm within 6 h after rupture. Although we performed regression analyses with adjustments for variables such as WFNS score, and additional analyses to account for bias by indication in patient with rebleeding, this did not change our findings.

We conclude that our results do not support a strategy in which major efforts are made to treat every patient with a ruptured aneurysm within 6 h after rupture. Since this was a non-randomized comparison and the data even suggest that aneurysm treatment within 6 h results in worse outcomes, we recommend further studies on this subject. A randomized controlled trial will not be easy to perform, but should be considered.
